# Supplementary material for: Generating ring-shaped engineered heart tissues from ventricular and atrial human pluripotent stem cell-derived cardiomyocytes
Source: Nat Commun. 2020 Jan 7;11:75. doi: 10.1038/s41467-019-13868-x (PMC6946709; doi:10.1038/s41467-019-13868-x)
Supplement: Supplementary file 1 — Supplementary Information [file 41467_2019_13868_MOESM1_ESM.pdf]

SUPPLEMENTARY INFORMATION

Generating Ring-Shaped Engineered Heart Tissues from Ventricular and Atrial Human Pluripotent Stem Cell-Derived Cardiomyocytes

I. Goldfracht et al.

Supplementary Figures

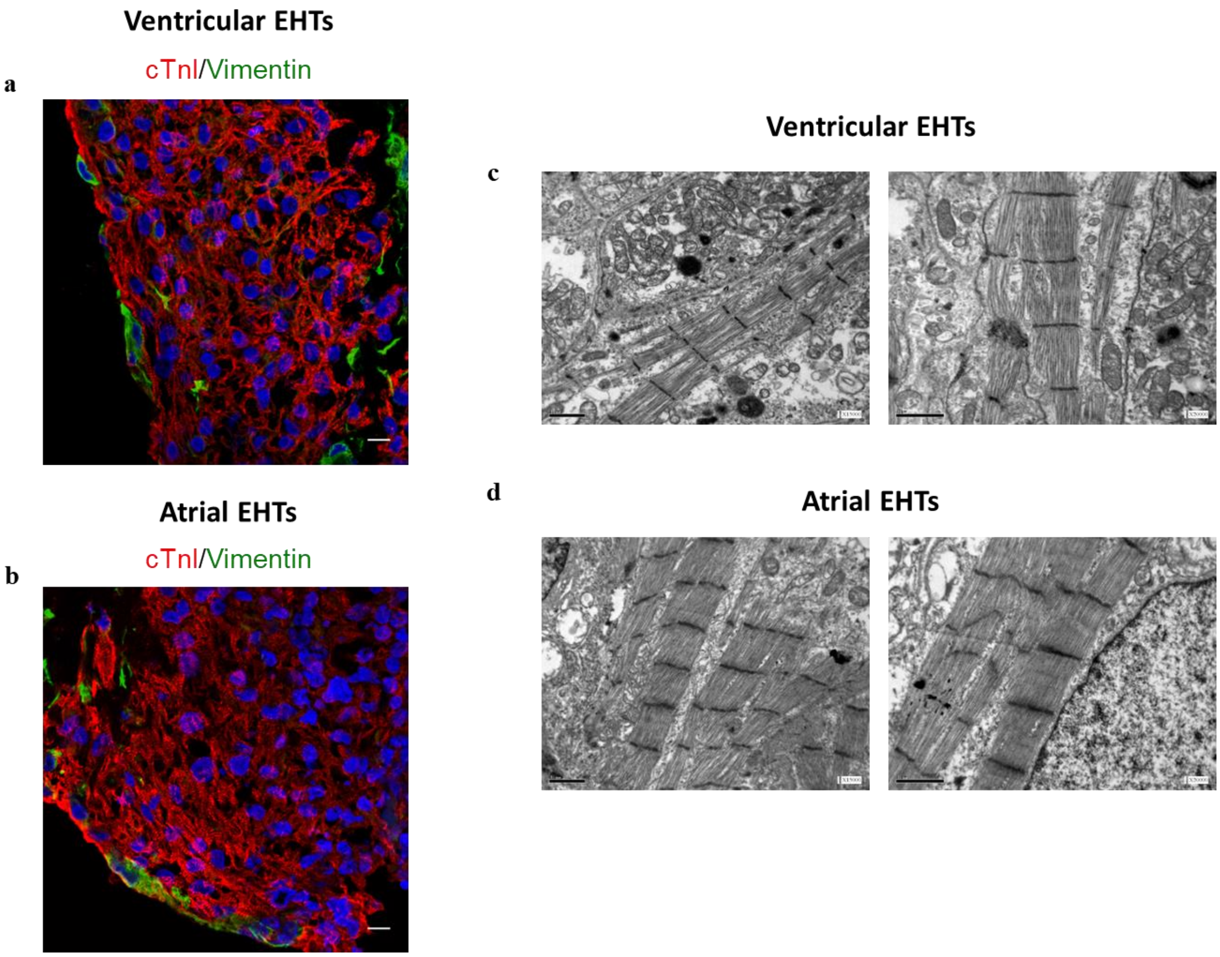

Fig. 1. Ultrastructural characterization of the chamber-specific EHTs.

a-b Co-immunostaining of 30d ventricular **(a)** and atrial **(b)** EHTs for cTnI with the fibroblast marker vimentin. Nuclei were stained with DAPI. Scale: 10µm. 8 images were like the image presented here, out of 9 experiments performed. **c-d** Transmission electron microscopy (TEM) images of the chamber-specific EHTs. Organized sarcomeres, Z-lines and mitochondria are noticeably present in both ventricular **(c)** and atrial **(d)** EHTs. Scale: 1µm. 39 images were like the image presented here, out of 50 experiments performed.

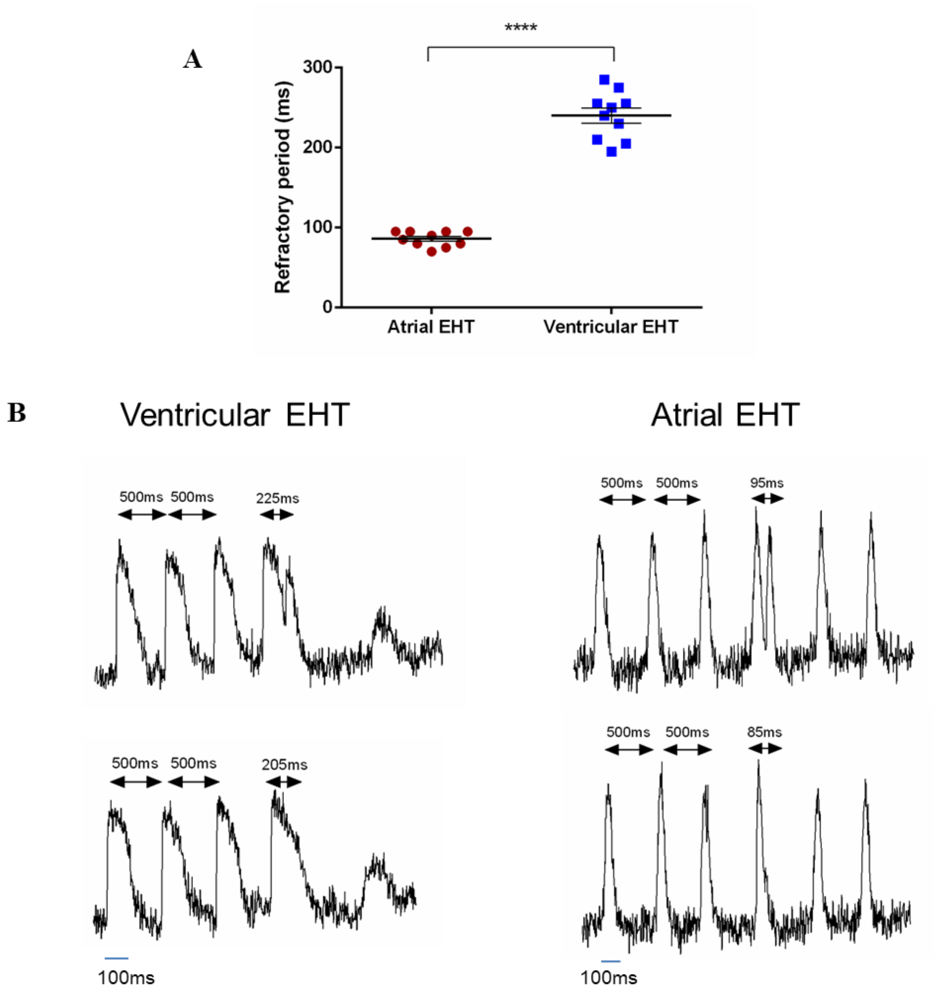

Fig. 2. Refractory period of chamber-specific EHTs.

a Refractory period of atrial and ventricular EHTs. ****p<0.0001, unpaired two-tailed t test, n=10, biologically independent samples. Values are expressed as mean±SEM. **b** Representative images showing action potential recordings of ventricular and atrial EHTs. The constructs were paced in 2 Hz for 10 sec and underwent a premature stimulation. In each chamber-specific EHT the last cycle length that resulted in AP stimulation is shown. In further decreasing of cycle length, no AP could be stimulated.

a

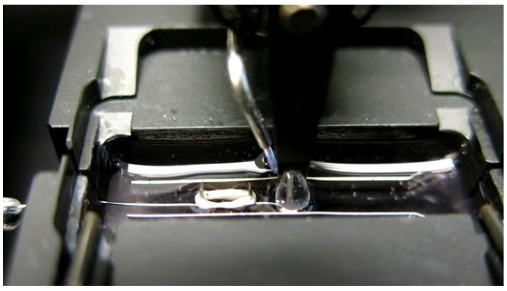

b

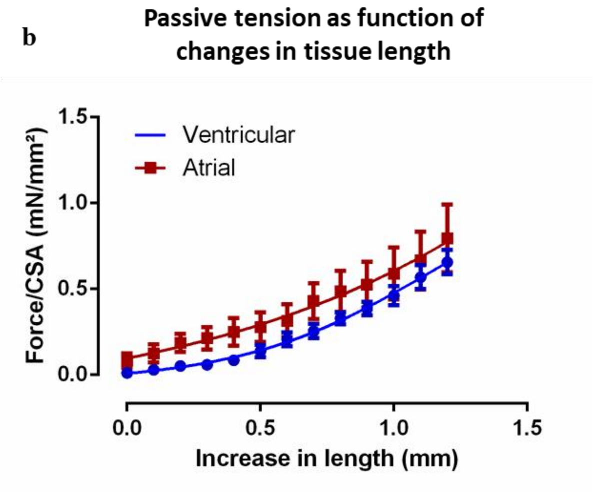

Fig. 3: Passive tension in the chamber-specific EHTs.

a Representative image of the EHT in the experimental setup used for gradual stretching of the tissues and for measurements of the generated passive and active forces. **b** Passive tension measurements of the atrial and ventricular EHTs, normalized to cross sectional area (CSA) and plotted against the change in length (n = 6, biologically independent samples). Values are expressed as mean±SEM.

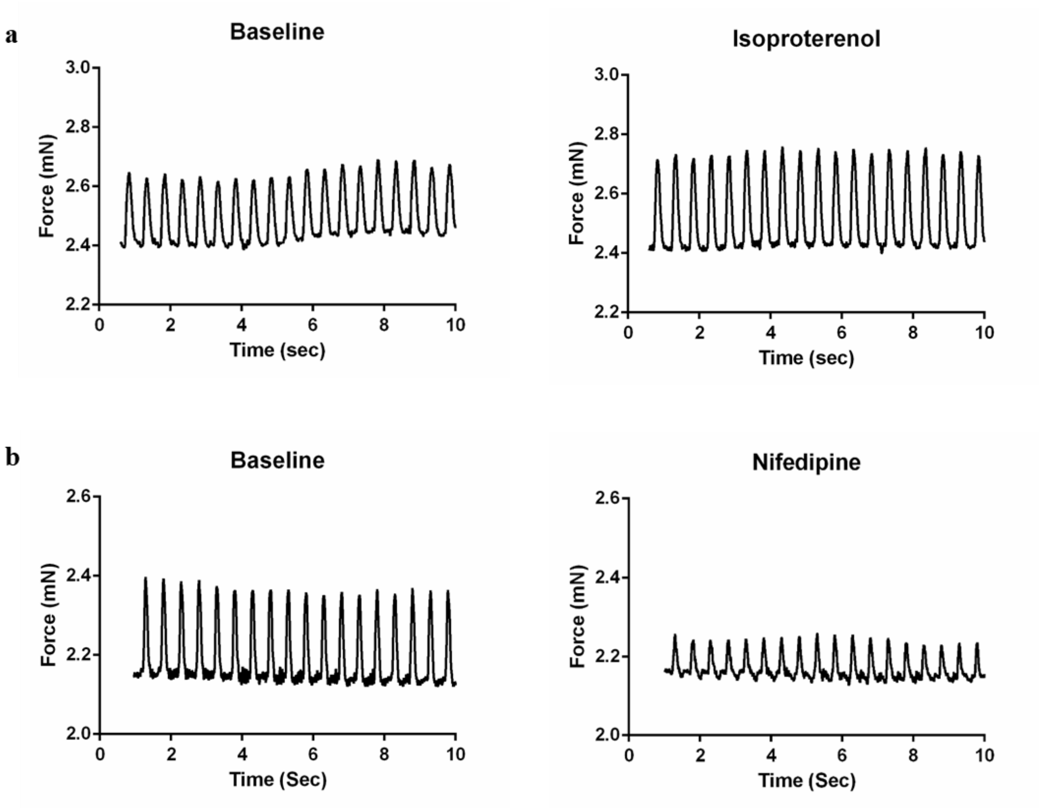

Fig. 4. Pharmacological effects on contractile force measurements in the atrial EHTs.

a-b Examples of measured contraction forces of the atrial-EHTs following application of 10 µM of isoproterenol (**a**) or of 0.1 µM nifedipine (**b**). Note the resulting positive and negative ionotropic effects respectively. All recordings were performed at a stimulation frequency of 2 Hz.

Supplementary Table 1: Primers used for qPCR gene expression analysis

Primer set (all human)	Primer	Sequence (5'-3')
GAPDH	Fwd	CAGCCTCAAGATCATCAGCAATG
	Rev	CCATCCACAGTCTTCTGGGTG
NPPA	Fwd	ACAGGATTGGAGCCCAGAG
	Rev	GGAGCCTCTTGCAGTCTGTC
KCNA5	Fwd	CTGCTCATCTTCTTCCTCTTCATCG
	Rev	TCAGGGATGCTAGAGAAATGGGTTC
KCNJ3	Fwd	GCACGCGGTGATCTCCATGA
	Rev	ACCCTCAGGTGTCTGCCGA
MYL7	Fwd	AGGAGTTCAAAGAAGCCTTCAGCT
	Rev	AGCATGGCGTCCAGCTCCTC
NR2F2	Fwd	AGCAAGTGGAGAAGCTCAAGGC
	Rev	TGGGCTACATCAGAGAGACCAC
GJA5	Fwd	AGATCATCTTCGTCTCCACGCC
	Rev	CGGGTACTCGTAAGAGCCAGAG
MYL2	Fwd	TCTGAGAGACACCTTTGCTGCC
	Rev	GGGTCCGCTCCCTTAAGTTTCT
GJA1	Fwd	AGATGAGCAGTCTGCCTTTCGT
	Rev	TGAGCCAGGTACAAGAGTGTGG
MYH7	Fwd	AAAGAGGCGCTAGAGAAGTCCG
	Rev	CAGCATCTGCCAGGTTGTCTTG
TNNT2	Fwd	CTGGCCATTGACCACCTGAATG
	Rev	CTGCTGCTTGAACTTCTCCTGC
HEY2	Fwd	CTGAGTTGAGAAGACTTGTGCCAA
	Rev	TGGCAAGAGCGTGTGCGTCAAA
KCNJ2	Fwd	AACAGTGCAGGAGCCGCTTT
	Rev	ATCCACCGCCAGCGAATGTC

**Primer set (all human)**	**Primer**	**Sequence (5'-3')**
TNNT1	Fwd	GATCTGCGGGCCAACCTCAA
	Rev	GTCGGAGACTTGGCGGCAT
