# Supplementary material for: Generating ring-shaped engineered heart tissues from ventricular and atrial human pluripotent stem cell-derived cardiomyocytes
Source: Nat Commun. 2020 Jan 7;11:75. doi: 10.1038/s41467-019-13868-x (PMC6946709; doi:10.1038/s41467-019-13868-x)
Supplement: Supplementary file 10 — Description of Additional Supplementary Files [file 41467_2019_13868_MOESM10_ESM.pdf]

Title: Supplementary Movie 1:
Description: Spontaneously contracting atrial and ventricular EBs at day 20 of differentiation.

Title: Supplementary Movie 2:
Description: Representative spontaneous contraction of atrial and ventricular EHTs suspended on silicon stretchers**.**

Title: Supplementary Movie 3:
Description: Activation wave-front propagation in ventricular EHTs during spontaneous rhythm

Title: Supplementary Movie 4:
Description: Spontaneous development of different types of arrhythmias in the atrial EHTs.

Title: Supplementary Movie 5:
Description: Conversion of arrhythmias in atrial EHTs into normal rhythm using field stimulation.

Title: Supplementary Movie 6:
Description: Pharmacological conversion of arrhythmias in the atrial EHTs into normal rhythm by 10 µM flecainide or 30 µM verankalant.
